# The combined effect of cadmium and copper induces bioaccumulation, and toxicity and disrupts the antioxidant enzymatic activities of goldfish (*Carassius auratus*)

**DOI:** 10.1016/j.toxrep.2025.101972

**Published:** 2025-02-28

**Authors:** Zeeshan Ali, Nadia Sher, Ijaz Muhammad, Gul E Nayab, Abdulaziz Alouffi, Mashal M. Almutairi, Ijaz Khan, Abid Ali

**Affiliations:** a State Key Laboratory of Marine Resource Utilization in South China Sea, School of Marine Science and Engineering, Hainan University, China; b Department of Zoology, Abdul Wali Khan University, Mardan 23200, Pakistan; c Department of Chemistry, Islamia College University, Peshawar, Pakistan; d King Abdulaziz City for Science and Technology, Riyadh 12354, Saudi Arabia; e Department of Pharmacology and Toxicology, College of Pharmacy, King Saud University, Riyadh 11451, Saudi Arabia

**Keywords:** Gold Fish, Bioaccumulation, Cadmium, Copper, Tissues, Blood

## Abstract

An aquatic environment polluted with cadmium (Cd) and copper (Cu) has threatened fish health and adversely affected the aquaculture industry's sustainable development. The study revealed that exposure to Cd and Cu caused significant bioaccumulation in goldfish tissues, particularly in gills, intestine, and muscles. The bioaccumulation of these heavy metals increased with exposure time, with the highest levels recorded after 96 hours. This prolonged exposure led to a range of adverse effects on the fish's physiological functions. Hematological parameters, including white blood cells, red blood cells, and platelets, decreased significantly, indicating a compromised immune system. Conversely, some hematological parameters, such as hemoglobin and hematocrit, increased with exposure, suggesting a potential compensatory response. Biochemical parameters, including serum glutamic pyruvic transaminase, blood urea, and serum triglycerides, also increased with exposure, indicating liver damage and disrupted metabolic functions. Furthermore, the study found that antioxidant enzymes, including superoxide dismutase, catalase, and ascorbic acid, decreased significantly, while malondialdehyde concentration increased, indicating oxidative stress and lipid peroxidation. These findings collectively suggest that Cd and Cu exposure can cause significant toxicity in goldfish, affecting their hematological, biochemical, and enzymatic functions, and highlighting the need for further research into the effects of these heavy metals on aquatic organisms.

## 1 Introduction

Aquaculture has always been highly valuable in sustainable nutrition and employment. Fisheries contribute substantially to addressing growing food demand and nutritional deficiencies, being the best source of proteins, vitamins, minerals, and omega-3 fatty acids [1]. It is a source of employment, uplifting livelihood and human socioeconomic well-being [2]. As inhabitants of aquatic ecosystems, fish are profoundly prone to heavy metals as stable and high-potential contaminants [3], [4], [5], [6]. Heavy metals in aquatic ecosystems threaten fish health and aquaculture sustainability, accumulating through gills, skin, [7] and food ingestion, [8]. The severity of these impacts depends on factors like concentration, exposure time, fish size, and species, and can have far-reaching consequences for aquaculture, including reduced fish populations, decreased productivity, and compromised food safety [9] by harming growth, physiology, and reproduction, emphasizing the need to monitor and mitigate pollution for sustainable aquaculture.[5], [10], [11], [12], [13]. Aquatic ecosystems are increasingly contaminated with heavy metals, posing a significant threat to fish populations. Heavy metals such as Cd, Cu, nickel (Ni), chromium (Cr), arsenic (As), lead (Pb), selenium (Se), zinc (Zn), and mercury (Hg) can accumulate in fish bodies, causing harm. [14], [15]. Among these, Cd is particularly concerning, being a non-essential and highly toxic metal that disrupts enzymatic functions and induces oxidative stress [16] while Cu have also devastating effects on fish health [17]. In addition, these metals can impair organ function, disrupt physiological processes, hinder growth [17], [18], induce anemia [19], [20], damage reproductive organs, and decrease fertility. [10], [21]. Moreover, excessive Cu intake can lead to reduced appetite, distorted reproductive organs, and decreased gonadosomatic index, fecundity, fertilization, and hatching rate [22], [23]. Assessing hematological indices is crucial in evaluating the extent of damage caused by these toxic elements on fish blood cells. [24]. Changes in fish biochemical and hematological characteristics can indicate structural and physiological changes in response to heavy metal exposure. Therefore, analyzing these characteristics are essential for understanding the impact of environmental pollution on fish health. Hematological and biochemical assessments can serve as valuable indicators of physiological imbalance, enabling researchers to evaluate the effects of heavy metal exposure on fish populations. [25].

*C. auratus*, a widely cultivated freshwater fish species, is particularly vulnerable to heavy metal contamination, specifically Cu and Cd, in freshwater ecosystems. As a dominant species in lake fish culture, its high abundance and adaptability to changing environmental conditions make it an important sentinel species for monitoring heavy metal pollution. The species' sensitivity to Cd and Cu contamination is of concern, as these metals can accumulate in its tissues and have toxic effects, posing a significant threat to its populations and the overall ecosystem. [26]. As reported earlier, *the C. auratus* resulted in the potential enrichment of liver, gill, gut, and muscle with Cd and Cu treatment in a controlled incubational study. Exposure to increasing concentrations of Cd and Cu caused a collective and severe pathological impact on *C. auratus*, leading to widespread histopathological changes. The heavy metal contamination induces tissue damage, inflammation, and degeneration in various organs, including the liver, gills, gut, and muscles, ultimately compromising the overall health and well-being of the fish [27]. Despite the ecological importance of *C. auratus*, there is a significant knowledge gap regarding the combined effects of Cd and Cu on this species. To date, research has focused primarily on individual metal toxicity, leaving a critical need for comprehensive studies on the synergistic effects of Cd and Cu accumulation, as well as the resulting biochemical responses in fish exposed to these heavy metals over specific time periods. The present study focuses on investigating the impact of cadmium (Cd) and copper (Cu) on *C. auratus*, specifically examining the bioaccumulation of these heavy metals in various tissues and their effects on hematological and biochemical characteristics.

## 2 Material and methods

### 2.1 Ethical approval

All experimental techniques were approved by the Animal Research Ethics Committee of the Hainan Provincial Education Center for Ecology and Environment. Captured fish should be handled in a manner that minimizes pain, distress, suffering and unnecessary loss of external mucus or scales. The duration of handling and the experimental procedure should be kept to a minimum, to avoid unnecessary stress and exposure time [28].

### 2.2 Experimental fish and acclimatization

Fifty adults *C. auratus* were obtained from a local fish market in Haikou, Hainan, China. Following a three-week acclimatization period under standard laboratory conditions, the fish were fed twice daily, 2 % of their total body weight. After acclimatization, a random subset of 20 fish (body length, 13.7 ± 1.7 cm, body weight, 55.6 ± 7.4 g) was selected for the study using a stratified random sampling method to ensure representation across the size range. This approach ensured that the selected fish were representative of the overall population.

### 2.3 Preparation of stock solution for copper sulfate and cadmium chloride

Copper sulfate (CuSO_4_) and cadmium chloride (CdCl_2_) were used for the experiments. Stock solutions of 1000 mg/L (1000 ppm) were prepared by dissolving 1 g of CuSO_4_ and 1 g of CdCl_2_ in 1 L of distilled water, respectively. These stock solutions were stored at room temperature for further use. For the exposure experiments, sub-lethal concentrations of 1.15 mg/L CuSO_4_ and 0.93 mg/L CdCl_2_ were used, which were determined based on the 96-hour LC_50_ values of 2.7 mg/L for CuSO_4_ and 1.9 mg/L for CdCl_2,_ respectively.

### 2.4 Experimental design

Following the acclimatization period, the fish were randomly allocated into three separate glass tanks, each containing 130 L of water and a density of 20 fish per tank. These tanks were designated as control (Ck), Cu-treated (Cu), and Cd-treated (Cd). Fish were exposed to two sub-lethal concentrations of CuSO_4_ and CdCl_3_, precisely 1.15 mg/L to Cu and 0.93 mg/L to Cd, respectively. No chemicals were added to the control group. After 24 hours of exposure, five fish from each tank were selected and dissected on a cold plate, and their various organs (Gills, Intestine, Muscles, Skin, and Bones) were examined for bioaccumulation. The liver was also collected to assess antioxidant enzyme activity, each fish received three replicate samples of its organs. Blood samples were obtained for analysis of biochemical and hematological parameters. This procedure was repeated at 48 hours, 72 hours, and 96 hours, with five fish sacrificed from each tank every 24 hours up to the 96 hour mark. The 24 hours sampling interval was chosen to capture the dynamic changes in bioaccumulation and toxicity effects over time, allowing for the identification of potential time-dependent responses.

### 2.5 Measurement and analysis of bioaccumulation

*C. auratus* were dissected, and different organs were removed. 1.0 g of tissue was taken from each organ and dried in an oven at 120°C for 24 hours to achieve a constant weight. The dried samples were then subjected to microwave-assisted digestion using 20 mL of concentrated nitric acid (65 %) in a closed vessel at 180°C for 30 minutes. After digestion, the samples were centrifuged at 14,000 ×g for 20 minutes at 4°C to separate the supernatant. The resulting supernatant was filtered through a 0.45 μm membrane filter and diluted to a final volume of 50 mL with deionized water. The diluted samples were then analyzed for Cu and Cd concentrations using an atomic absorption spectrophotometer (AA-6300, Shimadzu, Japan).

### 2.6 Antioxidant enzyme analysis

The activities of liver superoxide dismutase (SOD), catalase (CAT), ascorbic acid (ASA) level, and malondialdehyde (MDA) content were detected following the instruction of the kits (Nanjing Jiancheng). The OD value was detected at the wavelength of 450 nm, 405 nm and 412 nm, respectively. One unit of SOD activity was calculated using the amount of superoxide dismutase required to inhibit the reduction of nitrobluete trazolium by 50 %. One unit of CAT activity was defined as the amount of CAT required to transform 1 μmol of H_2_O_2_ per min. In the presence of thiobarbituric acid, MDA started producing colored thiobarbituric-acid-reacting substances (TBARS) that were measured at 532 nm assayed according to the experimental procedures followed by [29], [30].

### 2.7 Determination of LOD and LOQ

The limit of detection (LOD) and limit of quantification (LOQ) were determined through a calibration curve generated from a series of standard solutions (0.1–10 mg/L) of Cu and Cd, with a correlation coefficient (R²) of 0.999. The calibration curve's slope was calculated using a linear regression model. The LOD was predicted from three times the standard deviation (SD) of ten blank replicates (deionized water) divided by the calibration curve's slope, while the LOQ was calculated from ten times the SD of ten blank replicates divided by the calibration curve's slope. [31].

### 2.8 Hematological and biochemical analysis

Blood samples from *C. auratus* were collected using heparin-coated syringes and centrifuged to separate plasma. Hematological parameters, including RBC count, hemoglobin content, and hematocrit value, were determined using standardized methods and instruments [32] [33]. Plasma biochemical parameters, such as glucose, total protein, lipid content, AST, and ALT activity, were analyzed using commercial assay kits and spectrophotometry, following established protocols [34] [35].

### 2.9 Statistical analysis

Statistical analysis was performed using SPSS version 23.0. One-way ANOVA was employed to determine significant differences among treatment groups. Post-hoc comparisons were made using Fisher's LSD test and Duncan's Multiple Range Test (DMRT) to separate means. Statistical significance was set at (p < 0.01).

## 3 Results and discussion

Heavy metals, particularly Cd and Cu, pose a significant threat to freshwater fish culture due to their high stability, bioaccumulation, and biomagnification characteristics [27]. While Cu is an essential nutrient for fish, its elevated concentrations above the recommended water quality standard of 2.9–31.4 μg/L can adversely affect fish health [36]. In contrast, Cd has no known metabolic function and can be toxic even at very low concentrations, with a recommended water quality standard of ≤ 0.25 μg/L, highlighting its potential as a significant pollutant. [37].

### 3.1 Bioaccumulation of heavy metals

**Fig. 1:**
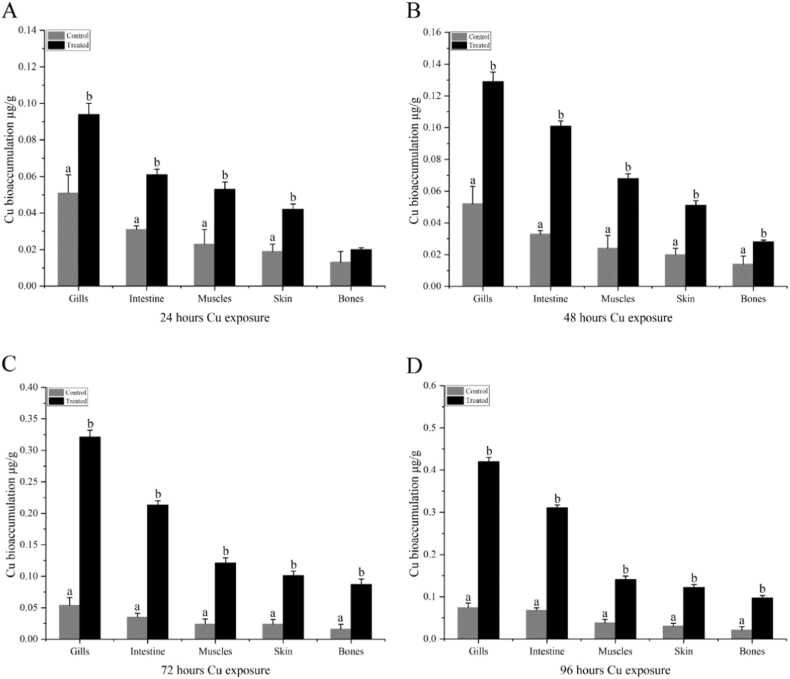
Copper bioaccumulation in the gills, intestine, muscles, skin, and bones of *C. auratus* during copper exposure (n = 5). Note: Ctrl (0.0 mg/L) and Cu (1.15 mg/L). (A): 24 hours, (B): 48 hours. (C): 72 hours, (D): 96 hours. Data are expressed as Mean ± S.E. Bar with different lowercase letters is significantly *(P < 0.01)* different between groups.

**Fig. 2:**
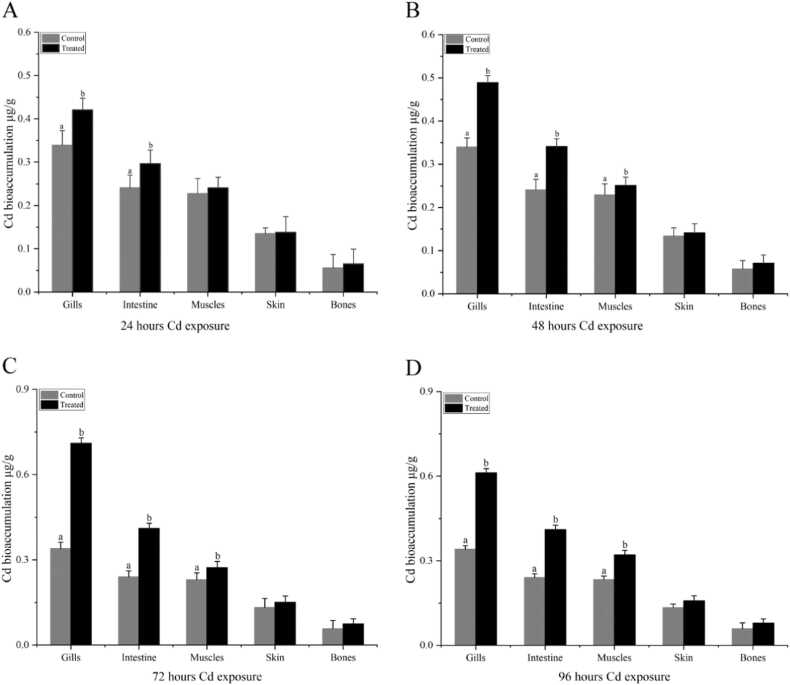
Cadmium bioaccumulation in the gills, intestine, muscles, skin, and bones of *C. auratus* during cadmium exposure (n = 5). Note: Ctrl (0.0 mg/L) and Cd (0.93 mg/L). (A): 24 hours, (B): 48 hours. (C): 72 hours, (D): 96 hours. Data are expressed as Mean ± S.E. Bar with different lowercase letters is significantly *(P < 0.01)* different between groups.

*C. auratus* a widely cultivated and ecologically successful freshwater fish, is commonly found in lake fish culture. Its ability to thrive in various environments, favored by climatic changes, makes it an ideal species to study the impact of heavy metal bioaccumulation, particularly in the context of altered ecosystems. [26]. Different reports showed that higher concentrations of heavy metals have been found in fish intestines than in muscles [38]. Our results showed that Cd (0.42 ± 0.02) and Cu (0.09 ± 0.02) accumulated significantly higher in gills compared to the lowest in bones as (0.06 ± 0.01) and (0.01 ± 0.04) respectively, after 24 hours of heavy metal exposure (Table 1). These concentrations exceed the recommended water quality standards indicating potential toxicity to *C. auratus*. Notably, the gill Cd concentration in our study is comparable to the range reported in previous studies on other fish species, such as (0.35–0.45 μg/g) in *Cyprinus carpio.* Fishes are exposed to heavy metals either through the food chain or from polluted water, and gills are the organs first to be contained with the metals found [39]. Further exposure to these metals showed a similar trend of Cd and Cu bioaccumulation in different organs and tissue of fish (gills, intestine, skin, and bones), indicating higher detection as 0.48 ± 0.04 and 0.12 ± 0.04, 0.71 ± 0.04 and 0.14 ± 0.04, 0.83 ± 0.04 and 0.32 ± 0.04 in gills after 48, 72 and 96 hours of exposure respectively. In comparison to *C. auratus,* where Cd and Cu accumulate in the order of Gills>intestine>muscles>skin>bones, other fish species exhibit distinct bioaccumulation patterns. For instance, Cd accumulation varies across species, with *Hydrocynus forskahlii* showing Gills>muscle>heart>kidney, *Clarias gariepinus* exhibiting Kidney>gills>heart, *Hydrocynusbebe occidentalis* displaying Gills>heart>muscle, and *Coregonus lavaretus* presenting Kidney>liver>gills>muscle. Similarly, Cu accumulation patterns differ among species, such as *Cyprinus carpio* (Gills>intestine>kidney>liver>muscle), *Pelteobagrus fulvidraco* (Liver>kidney>muscle>gills>intestine), and *Coregonus lavaretus* (Kidney>liver>gills>muscle). These variations highlight the species-specific nature of heavy metal bioaccumulation, underscoring the importance of studying individual species to understand their unique responses to metal exposure. [40]. Particularly, in *C. auratus,* it is reported that Cd retention is increased in the order kidney>intestine>liver>spleen>blood later on, leading to permanent damage of these tissues [41]. Consistent with our findings, Cu bioaccumulation in *C. auratus* exhibits a clear pattern with the highest concentrations found in the gills, followed by the intestine, muscles, skin, and bones., underscoring the vulnerability of gills and intestines to Cu toxicity. This observation aligns with the established understanding that Cu can damage organs and systems, including the gills, liver, kidney, immune system, and nervous system, ultimately compromising fish health. [42]. Copper has distinctive uptake mechanisms of a transmembrane protein and the Na^+^-uptake pathways located at branchial epithelial cells [43], [44]. The results showed that Cu and Cd accumulation in *C. auratus* increased significantly over time, with the highest concentrations found after 96 hours of exposure (Fig. 1 and Fig. 2). Specifically, the average accumulation of Cd and Cu in different organs and tissues decreased in the order of Gills>intestine>muscles>skin>bones, while the effect of exposure time decreased in the order of 96 hours> 72 hours> 48 hours > 24 hours. Notably, Cd accumulation increased by 11.4 %, 26 %, and 24.4 % after 24, 48, and 72 hours of exposure, respectively, while Cu accumulation increased by 28 %, 62.55 %, and 61.5 % over the same periods.

**Table 1:** Shows bioaccumulation in the gills, intestine, muscles, skin, and bones after 24, 48,72, and 96 hour exposure of *C. auratus* to copper and cadmium. All the values are expressed as (Mean ± SE) using Fisher’s LSD test. Presented values are Significant (≥ 0.1) at *p* ≤ 0.01.

**Time of exposure**	**Organs**	**Cadmium**		**Copper**	
		**Control**	**Treated**	**Control**	**Treated**
**24 hours**	Gills	0.34 ± 0.04	0.42 ± 0.02	0.05 ± 0.04	0.09 ± 0.02
	Intestine	0.24 ± 0.07	0.29 ± 0.04	0.03 ± 0.02	0.06 ± 0.04
	Muscles	0.22 ± 0.07	0.24 ± 0.02	0.02 ± 0.04	0.05 ± 0.02
	Skin	0.13 ± 0.04	0.13 ± 0.07	0.01 ± 0.02	0.04 ± 0.04
	Bones	0.05 ± 0.02	0.06 ± 0.01	0.01 ± 0.02	0.01 ± 0.04
**48 hours**	Gills	0.34 ± 0.04	0.48 ± 0.04	0.05 ± 0.04	0.12 ± 0.04
	Intestine	0.24 ± 0.07	0.34 ± 0.02	0.03 ± 0.02	0.10 ± 0.02
	Muscles	0.22 ± 0.07	0.24 ± 0.02	0.02 ± 0.04	0.04 ± 0.02
	Skin	0.13 ± 0.04	0.14 ± 0.02	0.01 ± 0.02	0.04 ± 0.01
	Bones	0.05 ± 0.02	0.07 ± 0.02	0.01 ± 0.02	0.02 ± 0.01
**72 hours**	Gills	0.34 ± 0.04	0.71 ± 0.04	0.05 ± 0.04	0.14 ± 0.04
	Intestine	0.24 ± 0.07	0.40 ± 0.04	0.03 ± 0.02	0.12 ± 0.05
	Muscles	0.22 ± 0.07	0.27 ± 0.07	0.02 ± 0.04	0.11 ± 0.02
	Skin	0.13 ± 0.04	0.15 ± 0.02	0.01 ± 0.02	0.09 ± 0.02
	Bones	0.05 ± 0.02	0.07 ± 0.09	0.01 ± 0.02	0.06 ± 0.04
**96 hours**	Gills	0.34 ± 0.04	0.83 ± 0.04	0.05 ± 0.04	0.32 ± 0.04
	Intestine	0.24 ± 0.07	0.61 ± 0.04	0.03 ± 0.02	0.21 ± 0.02
	Muscles	0.22 ± 0.07	0.32 ± 0.02	0.12 ± 0.02	0.02 ± 0.04
	Skin	0.13 ± 0.04	0.15 ± 0.04	0.11 ± 0.02	0.01 ± 0.02
	Bones	0.05 ± 0.02	0.08 ± 0.04	0.08 ± 0.02	0.01 ± 0.02

Similarly, the accumulation of Cd in *C. auratus* increased by 11.4 %, 26 %, and 24.4 % over 24, 48, and 72 hours of exposure, respectively. In comparison, Cu accumulation increased by 28 %, 62.55 %, and 61.5 % over the same periods. These findings suggest that *C. auratus* tends to accumulate Cu more rapidly than Cd, particularly in the gills and intestine, which showed the highest concentrations of both metals. The observed bioaccumulation patterns can be attributed to the unique physiological characteristics of *C. auratus*, such as its gill morphology and intestinal absorption mechanisms, which facilitate the uptake and storage of heavy metals. [45] However, heavy metals generally accumulate in the metabolically active tissues of the living body [46].

Reports about other heavy metals revealed that Cr accumulates in order of gills>intestine>skin [47] and Pb as gills>muscles>visceral [48]. Thus, the gill is a fair entry point for Cu entrance [49]. Due to the negative charge, surface potential attracts positively charged metals [50]. The intestine's central digestive tract contains heavy metals [51], while the skin has direct exposure to the environment, which makes it prone to metal accumulation [52]. Thus, the gill and intestine have a relatively higher potential for metal accumulation than muscle, skin, and bones.

### 3.2 Hematological indices

In a stress-exposed aquatic environment, hematopoietic evaluation is a strong indicator to measure the health status of fish exposed because of sensitivity to toxicities [29]. Heavy metal accumulation in the fish bodies causes significant hematological changes through dysfunction of important organs like the liver, kidney, and gills having adverse effects on the physiology of fish and retarded growth as well [17], [18], [53]. Specifically, Cd caused changes in hematological indices by creating an irregularity in iron metabolism, causing fish anemia [19], [20] and inhibiting antioxidant enzymes in animals [54]. Similarly, excessive Cu intake by fish reduce food consumption which causes growth retardation [55]. Thus, the evaluation of hematological indices is the best way for the expression of the health status of fish. We recorded similar findings in response to Cd and Cu treatment to fish under controlled conditions for 96 days of study (Table 2). Results revealed that the white blood cells (WBC), red blood cells (RBC), and platelets (PLT) were significantly decreased to 69.94 ± 0.06, 1.68 ± 0.01 and 16.84 ± 0.06 at 24 hours after Cd and Cu exposure compared to control as 124 ± 0.047, 2.78 ± 0.01 and 20.92 ± 0.18 respectively. In the case of hemoglobin (HB) and hematocrit (HCT), values were recorded as lowest at 6.8 ± 0.04 and 16.36 ± 0.01 after 72 hours and 96 hours of the application of the treatment compared to 12.06 ± 0.17 and 30.8 ± 0.12 at the initiation of study for both respectively. However, an opposite trend was recorded in the case of mean corpuscular volume (MCV), mean corpuscular hemoglobin (MCH), and mean corpuscular hemoglobin concentration (MCHC), showing a significant increase from 112.3 ± 0.36, 32.76 ± 0.15 and 30.24 ± 0.15 (at start of the study) to 121.8 ± 0.07, 43.7 ± 0.28 and 41.22 ± 0.01 at 72 hours, 24 hours and 41 hours of heavy metal exposure respectively. Thus, the results revealed that the Cd and Cu stress to *C. auratus* reduced WBC, RBC, PLT, HCT, and HB while resulting in high blood MCV, MCH, and MCHC. Our previous study on *C. carpio* showed that PLT and WBC decreased, whereas MCH and MCHC increased with exposure to manganese and chromium [56]. Naz et al. expressed that Cu treatment to *Labeo rohita* caused a potential decrease in RBC, HCT, and HB and a small but non-significant reduction in MCH and MCHC [57]. Exposure of Cd to fish above dietary level causes impairment in erythropoiesis, life span reduction of erythrocytes, shift in equilibrium of osmotic pressure in erythrocytes, and plasma electrolyte metabolism [58]. As reported earlier, Cd stress potentially reduced hematological indices in *C. auratus gibelio* [29]. Work on larvae of *C. auratus* confirmed that Cu stress deformed the body and caused high mortality [59]. Similarly, Cd stress to *Paralichthys olivaceus* effectively reduced HCT and HB [60] due to Cd toxicity and impaired hematopoietic status, leading to severe anemia [29]. In the case of *C. auratus gibelio,* Cd exposure leads to HCT and HB reduction through erythrocyte lysis [61] and inhibition of hemoglobin synthesis leading to anemia [58]. The previous reports also showed that Cd exposure significantly decreased RBCs, HB, and HCT in *Oreochromis niloticus* [58].

**Table 2:** shows the hematological parameters of both control and treated *C. auratus* after an exposure time of 24, 48, 72, and 96 hours to combine the effect of copper and cadmium. All the values are expressed as (Mean ± SE) using Fisher’s LSD test. Presented values are Significant (≥ 0.1) at *p* ≤ 0.01.

**Hematological Indices**	**Control group**	**Treated group**			
		**24 hours**	**48 hours**	**72 hours**	**96 hours**
White Blood Cells	124 ± 0.47	69.94 ± 0.06	89.07 ± 0.06	81 ± 0.41	74.66 ± 0.27
Hemoglobin (HBG)	12.06 ± 0.17	6.92 ± 0.05	8.94 ± 0.01	6.8 ± 0.04	7.92 ± 0.15
Red Blood Cells (RBCs)	12.06 ± 0.17	1.68 ± 0.01	3.74 ± 0.08	3.07 ± 0.03	2.00 ± 0.047
Hematocrit (HCT)	30.8 ± 0.12	18.99 ± 0.03	22.53 ± 0.03	22.05 ± 0.10	16.36 ± 0.01
Mean corpuscular volume (MCV)	112.3 ± 0.36	113.98 ± 0.02	105.35 ± 0.19	121.8 ± 0.07	101.01 ± 0.46
Mean corpuscular hemoglobin (MCH)	32.76 ± 0.15	43.70 ± 0.28	41.22 ± 0.10	38.9 ± 0.09	40.07 ± 0.03
Mean corpuscular hemoglobin concentration (MCHC)	30.24 ± 0.15	32.21 ± 0.05	41.22 ± 0.01	33.5 ± 0.09	39.92 ± 0.01
Platelets (PLT)	20.92 ± 0.18	16.84 ± 0.06	37.21 ± 0.04	26.59 ± 0.24	24.63 ± 0.25

### 3.3 Changes in biochemical characteristics

A shift in biochemical reactions in the fish body and changes in fish proteins could be a valuable bio-indicator for its physiological functions [62]. Variation in enzymatic activities could be a tool and a promising biomarker for metabolic body functions of fish, like hypoxic conditions, reduced antioxidant mechanisms, and tissue or cell destruction [63]. Our present study results (Table 3) indicated that the serum glutamic pyruvic transaminase (SGPT), blood urea, serum triglycerides, high-density lipoprotein (HDL), serum albumin, serum uric acid, and serum alkaline PO_4_ were recorded at 26.6 ± 0.27, 5.66 ± 0.27, 213.6 ± 0.27, 1.91 ± 0.04, 1.33 ± 0.02 and 209.6 ± 0.27 in control (no addition of Cd and Cu) which significantly increased to highest, i.e., 46.6 ± 0.27, 16.07 ± 009, 247.6 ± 0.27, 3.91 ± 0.09, 5.03 ± 0.02 and 270.6 ± 0.27 respectively at 96 hours of heavy metal exposure. Similarly, serum cholesterol, low-density lipoprotein, and lactate dehydrogenase (LDH) increased from 193.3 ± 0.27, 122 ± 0.47 and 1166 ± 0.47 in control to highest as 350 ± 0.47, 250.6 ± 0.27 and 1261.3 ± 0.27 at 24 hours while serum glutamic-oxaloacetic transaminase (SGOT) from 7.6 ± 0.27–213 ± 0.27 at 72 hours of Cd and Cu exposure to fish. In the case of serum creatinine, it decreased from the highest 0.84 ± 0.01 in control to 0.13 ± 0.01 at 72 hours of Cd and Cu treatment to *C. auratus*. It was also noticed that SGPT (25.31 ± 0.08), serum cholesterol (178.6 ± 0.27), and LDH (1121.3 ± 0.27) were found lowest at 24, 96 and 72 hours of metal exposure. Our results suggested that metal stress increased SGOT and SGPT levels, which showed hepatic damage. Enzymes like GOT and GPT present hepatocytes and function in the metabolism of amino acid and protein metabolism [29]. They are indicators of liver damage because, in such conditions, these enzymes move into the bloodstream [64]. Thus, the higher transaminase concentration indicates protein breakdown, which is necessary to deal with energy demand under such conditions [65].

**Table 3:** shows the biochemical parameters of both control and treated *C. auratus* after an exposure time of 24, 48, 72 and 96 hours to combine the effect of copper and cadmium. All the values are expressed as (Mean ± SE) using Fisher’s LSD test. Presented values are Significant (≥ 0.1) at *p* ≤ 0.01.

**Biochemical parameters**	**Control group**	**Treated group**			
		**24 hours**	**48 hours**	**72 hours**	**96 hours**
Serum Glutamic pyruvic transaminase SGPT	26.6 ± 0.27	25.31 ± 0.08	39.61 ± 015	40.6 ± 0.27	46.6 ± 0.27
Blood Urea	5.66 ± 0.27	9.06 ± 0.06	7.51 ± 0.05	15.34 ± 0.02	16.07 ± 0.09
Serum Creatinine	0.84 ± 0.01	0.38 ± 0.04	0.20 ± 0.02	0.13 ± 0.01	0.46 ± 0.02
Serum Triglycerides	213.6 ± 0.27	240 ± 0.47	225.3 ± 0.27	230 ± 0.47	247.6 ± 0.27
Serum Cholesterol	193.3 ± 0.27	350 ± 0.47	230.6 ± 0.27	185.3 ± 0.27	178.6 ± 0.27
High-density lipoprotein (HDL)	26.6 ± 0.27	38.49 ± 0.24	38.45 ± 0.11	41.32 ± 0.01	42.37 ± 0.01
Low-density lipoprotein	122 ± 0.47	250.6 ± 0.27	128.6 ± 0.27	131.16 ± 0.13	123.16 ± 0.13
Serum Glutamic-oxaloacetic transaminase SGOT	7.6 ± 0.27	15.6 ± 0.27	18.6 ± 0.27	21.3 ± 0.27	10.5 ± 0.23
Lactate dehydrogenase (LDH)	1166 ± 0.47	1261.3 ± 0.27	1248 ± 0.47	1121.3 ± 0.27	1210.3 ± 0.27
Serum Albumin	1.91 ± 0.04	2.97 ± 0.04	3.16 ± 0.01	3.91 ± 0.09	3.91 ± 0.09
Serum Uric Acid	1.33 ± 0.02	4.95 ± 0.01	3.72 ± 0.04	4.15 ± 0.07	5.03 ± 0.02
Serum Alkaline PO4	209.6 ± 0.27	212.6 ± 0.27	231.3 ± 0.27	251.6 ± 0.27	270.6 ± 0.27

### 3.4 Antioxidant enzymatic activities

**Fig. 3:**
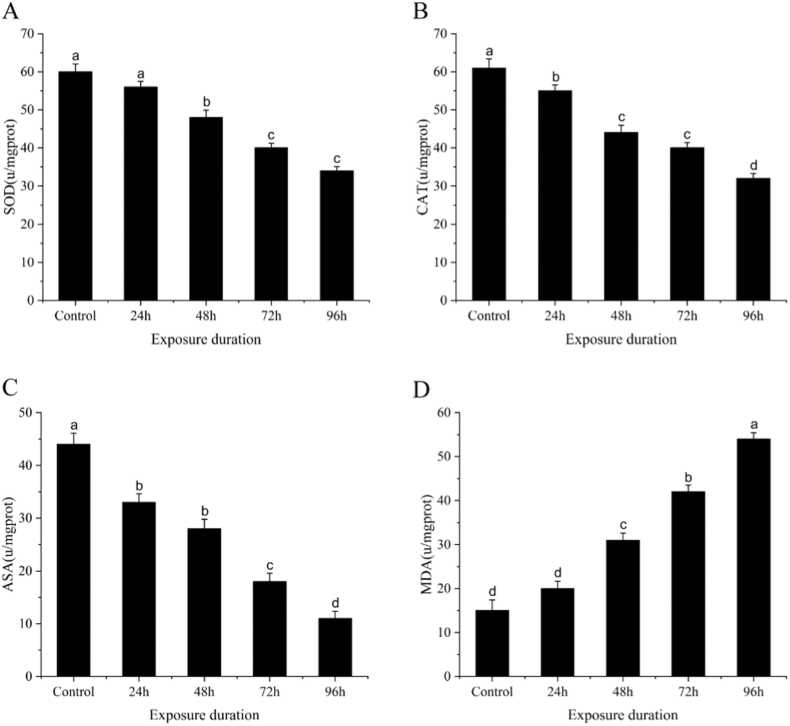
The activity of antioxidant enzymes in the liver of *C. auratus* during copper and cadmium exposure (n = 5) (A): SOD, (B): CAT, (C): ASA, (D) MDA. Bar with different lowercase letters is significantly *(P < 0.01)* different between groups.

Assessment of antioxidant responses evaluates oxidative stress in tissues [66]. Exposure of heavy metals to an elevated level undergoes oxidative stress, resulting in reactive oxygen species (ROS) being produced in excess, ultimately damaging tissue by oxidizing lipids or DNA [67]. In response to oxidative stress, antioxidant reactions occur in the body, thereby balancing ROS generation and antioxidant enzymes [68]. These enzymes named mainly as SOD, CAT, glutathione peroxidase (GPX), and glutathione reductase (GSR), mainly expressed in tissues where the antioxidant reaction occurs like liver tissues [69]. In our study, we examined that antioxidant enzymes, including SOD, CAT, and ASA, in the liver of *C. auratus,* decreased significantly with the exposure of the increasing metal. At the same time, MDA remarkably increased till 96 hours of Cd and Cu exposure to fish (Fig. 3). In fish, SOD and CAT are the first antioxidant defense system as SOD catalyzes the detoxification of the O_2_ radical to H_2_O_2_ and converted by CAT to H_2_O and O_2_. As a result of oxidative stress, enzyme activities are reduced by induction of ROS production. Following our findings, results have been reported earlier in the liver of *C. carpio* [70], Zebrafish [71], and *Platichthys stellatus* [72]. The MDA level also increased with Cd and Cu exposure time to fish. The MDA is an indicator of lipid peroxidation within cellular organelles and is reported to be elevated in cellular organelles in response to external stress stimuli [73].

## 4 Conclusions

In conclusion, this study reveals the alarming impact of Cd and Cu pollution on the health of *C. auratus*, an important species in aquaculture. The findings demonstrate significant bioaccumulation of Cd and Cu in fish tissues, accompanied by detrimental effects on hematological, biochemical, and enzymatic functions. The observed disruptions in blood parameters, liver function, and antioxidant defenses underscore the severity of heavy metal toxicity in fish. These results sound a warning bell for the aquaculture industry, emphasizing the need for stringent regulations and monitoring of heavy metal pollution to safeguard fish health and ensure sustainable aquaculture practices.

## Ethics statement

The experimental animal ethics committee at the Hainan Provincial Ecological Environment Education Center (HNECEE2019–005) provided the guidelines for the animal procedure utilized in this work.

## Funding

The researchers supporting project number (RSP2025R494), King Saud University, Riyadh, Saudi Arabia.

## CRediT authorship contribution statement

**Almutairi Mashal M:** Data curation, Formal analysis, Visualization. **E Nayab Gul:** Investigation, Writing – review & editing. **Khan Ijaz:** Conceptualization, Methodology. **Alouffi Abdulaziz:** Data curation, Formal analysis, Visualization. **Ali Abid:** Supervision, Methodology, Conceptualization. **MUHAMMAD Ijaz:** Writing – original draft, Writing – review & editing. **Ali Zeeshan:** Writing – original draft, Writing – review & editing. **Sher Nadia:** Investigation, Writing – review & editing.

## Declaration of Competing Interest

The authors declare that they have no known competing financial interests or personal relationships that could have appeared to influence the work reported in this paper.

## Data Availability

Data will be made available on request.
